# Ecosystem respiration and its components in a rainfed spring maize cropland in the Loess Plateau, China

**DOI:** 10.1038/s41598-017-17866-1

**Published:** 2017-12-14

**Authors:** Xiang Gao, Xurong Mei, Fengxue Gu, Weiping Hao, Haoru Li, Daozhi Gong

**Affiliations:** Key Laboratory of Dryland Agriculture, Ministry of Agriculture of the People’s Republic of China, Institute of Environment and Sustainable Development in Agriculture, Chinese Academy of Agricultural Sciences, Beijing, 100081 China

## Abstract

We estimated ecosystem respiration (Re) and its components in a rainfed spring maize field in the Loess Plateau, China, during the growing seasons of 2012, 2013, and 2014 using measurements of eddy covariance and soil respiration (Rs). The multi-factor equation, which included photosynthetic active radiation, 5-cm soil temperature, 10-cm soil water content, and green leaf area index (GLAI), had goodness-of-fit values of between 0.81 and 0.94 for Re, autotrophic respiration (Ra), and above-ground autotrophic respiration (Raa), and goodness-of-fit values of between 0.50 and 0.67 for Rs, below-ground autotrophic respiration (Rab), and heterotrophic respiration (Rh). The highly significant linear correlations between gross primary production (GPP) and Re and its components indicate that GPP had a strong influence on Re and its components. The growing season Re was dominated by Ra (64–71%), which in turn was dominated by Raa (63–73%). Although Rs was mainly made up of Rh (56–61%), Rs resembled Rab more closely than Rh. The relationships between GLAI and Ra/Re and between GLAI and Rab/Rs were described by logarithmic equations with goodness-of-fit values of between 0.88 and 0.89 and between 0.77 and 0.84, respectively, indicating that GLAI controlled Ra/Re and Rab/Rs.

## Introduction

The amount of carbon released to the atmosphere by ecosystem respiration (Re) represents the second largest carbon flux after photosynthesis, and photosynthesis generally referred to as gross primary production (GPP)^1^. Small changes in either of these two fluxes may therefore have a significant effect on the atmospheric CO_2_ concentration^2^. In particular, Re increase with temperature could exert a positive feedback on the climate system, leading to a global warming acceleration^3^. Previous studies have suggested that Re is an important influence on the carbon balance in various ecosystems^2,4,5^. Therefore, to gain an improved understanding of the relationship between the terrestrial carbon balance and environmental influences, we need to ascertain how Re and its components are influenced by abiotic and biotic factors.

Ecosystem respiration can be partitioned into heterotrophic respiration (Rh) from microbial decomposition of residues and soil organic matter and autotrophic respiration (Ra) from plants, or partitioned into above-ground autotrophic respiration (Raa) from the canopy of plants and soil respiration (Rs). Soil respiration can be further partitioned into Rh and below-ground autotrophic respiration (Rab) from the roots of plants. Autotrophic respiration and Rh are usually supported by current photoassimilates and soil organic matter, respectively^6,7^, and both will increase under global warming, but Ra may also decrease because of global dimming (i.e. the gradual reduction in the amount of global irradiance at the Earth’s surface). In addition, different components of Re may have different sensitivities to the same environmental factors (e.g., temperature and soil moisture)^5,8^. Therefore, it is necessary to identify the relationships between Re and its components to improve our understanding of the mechanisms that control Re.

Although cropland accounts for about only 12% of the global land area^9^, carbon cycling in cropland can significantly affect the global carbon balance^10^. Of all land use types, CO_2_ emissions from cropland to the atmosphere are considered the highest^11^, and account for nearly 25% of the CO_2_ released globally from human sources^12^. Cropland also has great potential for carbon storage under appropriate management practices, such as no-tillage, straw return, manure, and mulching with plastic film^13–16^. Human activities, including irrigation, fertilizer, and carbon inputs (residue and manure), have more influence on agricultural ecosystems than on natural ecosystems, and may considerably influence Re and it components^7,17,18^. Cropland is characterized by rapid vegetation development, meaning that plant growth may have a stronger influence on Ra and Re than do environmental factors^2,19^. Maize, grown extensively at mid- and low-latitudes^18,20–22^, plays an important role in ensuring global food security. To date, Re and Rs, thought to be controlled by temperature and soil moisture^8,17,23,24^, have generally been examined separately in maize fields rather than together. Therefore, to improve our understanding of carbon cycling in maize fields, it is necessary to obtain detailed information about the mechanisms that control Re and its components.

Rainfed cropland comprises more than 80% of cultivated land worldwide^9^. The Loess Plateau in China is an important rainfed agricultural region that covers an area of ~9° latitude and ~11° longitude, and includes a range of climate types, including arid, semi-arid, and sub-humid^25^. Spring maize (*Zea mays L*.) is one of the most common grain crops in this region^26^, and temperature and precipitation are the most important environmental controls on its growth^27,28^. Warming and dimming trends, accompanied by a significant decrease in the average daily rainfall intensity and an increase in the number of consecutive dry days, have been reported for the Loess Plateau^29,30^. These changes in climate may have noticeable impacts on the growth of spring maize and the local carbon budget. To date, there have been no systematic studies of Re and its components in rainfed spring maize cropland in the Loess Plateau.

Eddy covariance techniques, combined with soil chamber measurements, are commonly used to measure Re and its components^2,31,32^. In this study, we used this approach to measure Re and its components during the growing seasons of 2012, 2013, and 2014 in a spring maize cropland in the Loess Plateau. The aims of this study were to (I) characterize seasonal variations in Re and its components, and determine the sensitivity of these variations to temperature; (II) investigate the controls on Re and its components, and examine how they are influenced by GPP; and (III) identify the relationships between Re, Ra, and Rs and their components, and assess the effects of plant growth on the partitioning of Re, Ra, and Rs.

## Results

### Environmental factors and GPP

**Figure 1:**
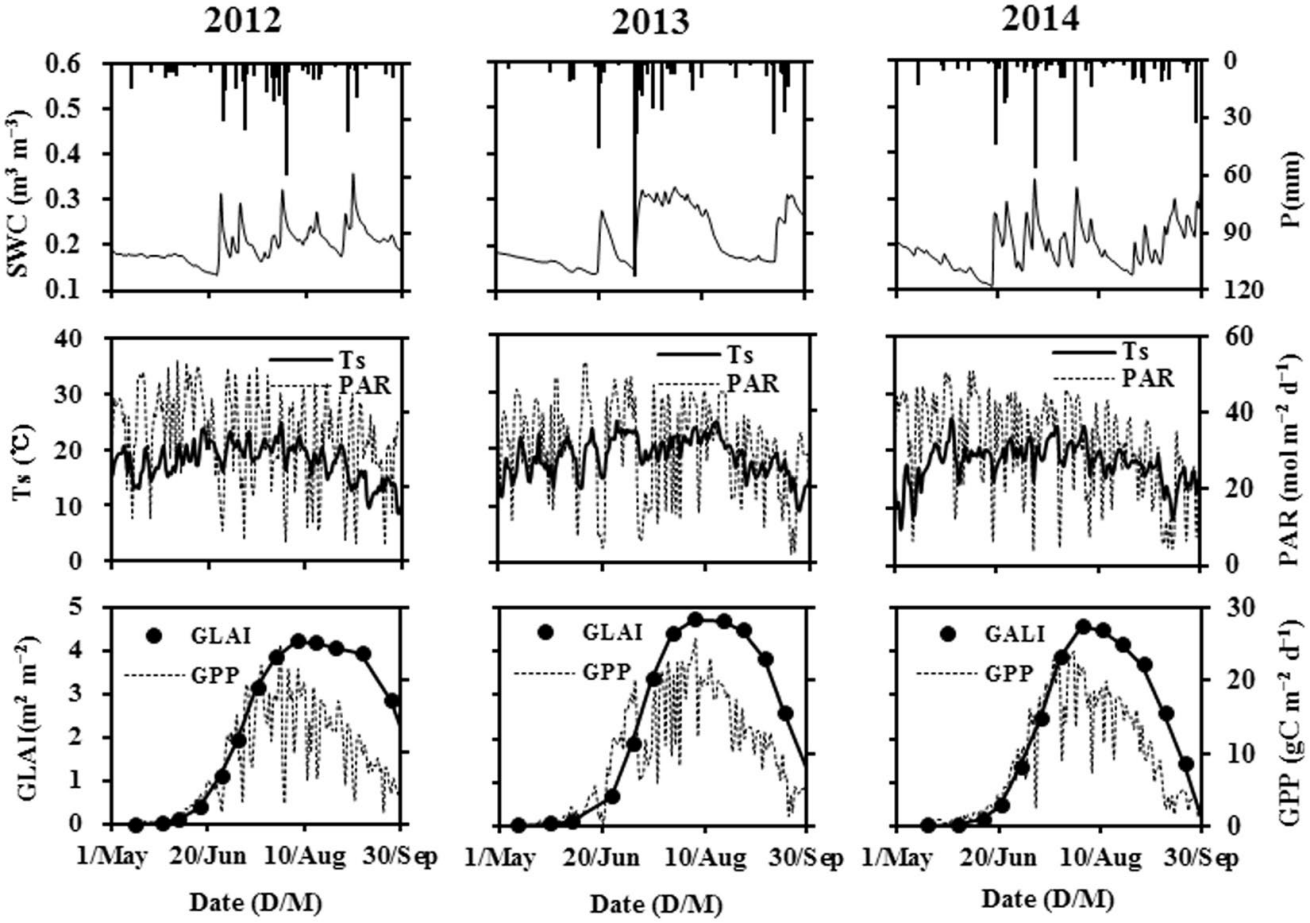
Dynamics of precipitation (P), 10-cm soil water content (SWC), 5-cm soil temperature (Ts), photosynthetically active radiation (PAR), green leaf area index (GLAI), and gross primary production (GPP) during the growing seasons of 2012, 2013, and 2014.

Seasonal variations in environmental factors and GPP during the three growing seasons are shown in Fig. 1. Precipitation amounts of 417, 497, and 411 mm were recorded during the growing seasons of 2012, 2013, and 2014, respectively. The 10-cm soil water content (SWC) increased suddenly following precipitation events and then decreased gradually. Cloudy and rainy days caused obvious fluctuations in the 5-cm soil temperature (Ts) and photosynthetically active radiation (PAR). The average growing season Ts and PAR reached maximum values of 20.03 °C and 33.97 mol m^−2^ d^−1^ in 2013 and 2012, respectively. The green leaf area index (GLAI) reached maximum values of 4.21, 4.72, and 4.58 m^2^ m^−2^ in early August of 2012, 2013, and 2014, respectively. The spring maize wilted rapidly because of northern corn leaf blight in the latter part of the 2013 and 2014 growing seasons. Gross primary productivity generally exhibited a mono-peak curve, and the average GPP reached a maximum of 10.02 g C m^−2^ d^−1^ during the 2013 growing season.

### Dynamics of Re and its components during the growth season

**Figure 2:**
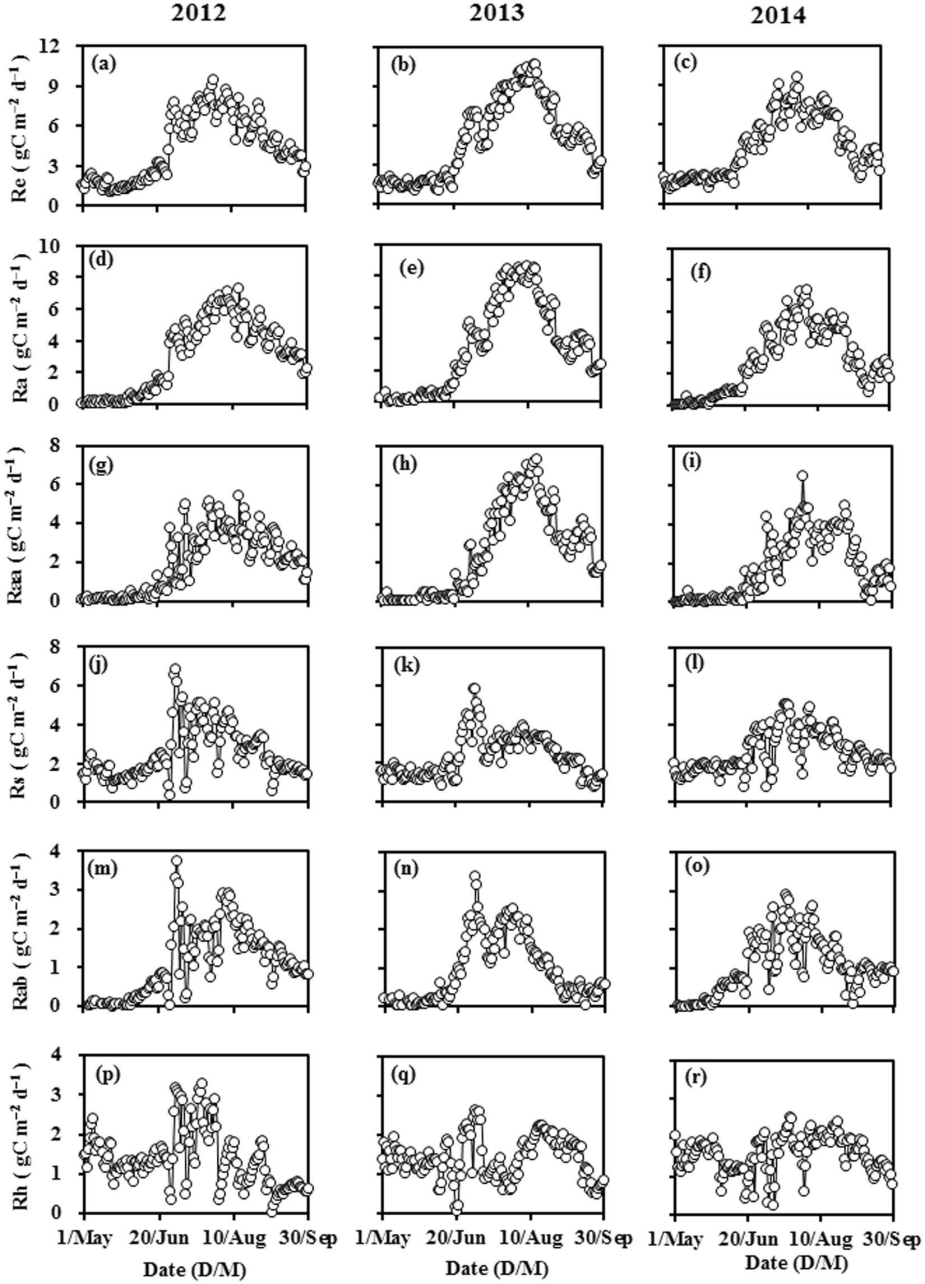
Dynamics of ecosystem respiration (Re), autotrophic respiration (Ra), above-ground autotrophic respiration (Raa), soil respiration (Rs), below-ground autotrophic respiration (Rab), and heterotrophic respiration (Rh) during the growing seasons of 2012, 2013, and 2014.

The daily Re, Ra, Raa, Rs, and Rab increased gradually at the start of the growing season and then decreased as the growing season progressed, whereas Rh followed a different pattern (Fig. 2). Even though Ts was relatively high in the early part of the growing season, the daily values of Ra, Raa, and Rab were close to zero because the spring maize was exceedingly small at that time. Maximum Ra and Raa values of 8.60 and 7.31 g C m^−2^ d^−1^, respectively, were measured in early August of 2013. Similarly to the daily Rs and Rh, the daily Re was between 1 and 2 g C m^−2^ d^−1^ in the early part of the growing season, and reached a maximum of 10.63 g C m^−2^ d^−1^ in early August of 2013. The daily Rs, Rab, and Rh were affected by precipitation, and decreased sharply, sometimes to zero, on rainy days. When the soil was dry, Rs, Rab, and Rh displayed a priming effect because of precipitation, and their values were much higher after precipitation than in the pre-precipitation period. When the soil was wet, the values of Rs and its components after precipitation only recovered to pre-precipitation levels.

### Drivers of Re and its components

#### Responses of Re and its components to environmental factors

**Figure 3:**
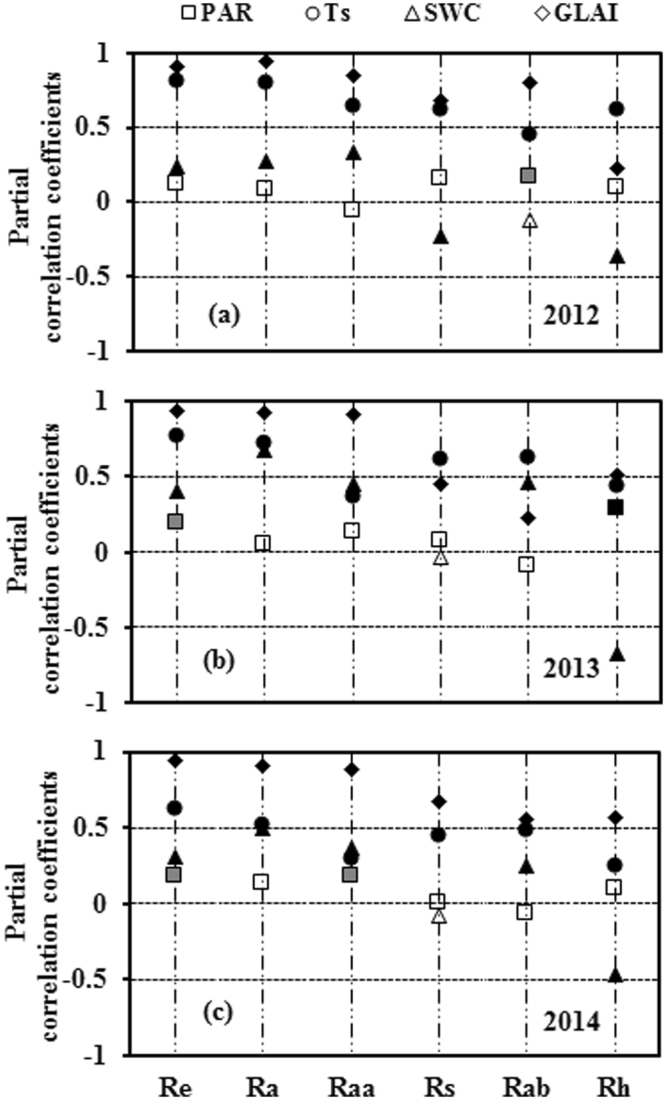
Partial correlation coefficients between ecosystem respiration (Re), autotrophic respiration (Ra), above-ground autotrophic respiration (Raa), soil respiration (Rs), below-ground autotrophic respiration (Rab), and heterotrophic respiration (Rh), and photosynthetically active radiation (PAR), 5-cm soil temperature (Ts), 10-cm soil water content (SWC), and the green leaf area index (GLAI) during the growing seasons of 2012, 2013, and 2014; The solid, gray, and open symbols represent results that are statistically significant at the p < 0.01, p < 0.05, and p > 0.05 levels, respectively (n = 153).

We performed a partial correlation analysis between Re and its components and environmental factors (PAR, Ts, SWC, and GLAI), as shown in Fig. 3. Values of PAR were significantly and positively correlated with Re (2013 and 2014), Ra (2014), Rab (2012), and Rh (2013). The positive correlations between Re and its components and Ts were highly significant, and Ts was the most important influence on Rs in 2012 and 2013, Rab in 2013, and Rh in 2014. The temperature sensitivity (Q_10_) values of Re and its components are listed in Table 1. Apart from Rab (2012) and Rs (2013 and 2014), the partial correlations between SWC and Re and its components were highly significant, and SWC was negatively correlated with Rh and Rs (2012). The GLAI was the most important influence on Re, Ra, and Raa; the positive relationships between GLAI and Rs, Rab, and Rh were also highly significant.

**Table 1:** Temperature sensitivity (Q_10_) of ecosystem respiration (Re), autotrophic respiration (Ra), above-ground autotrophic respiration (Raa), soil respiration (Rs), below-ground autotrophic respiration (Rab), and heterotrophic respiration (Rh) during the growing seasons of 2012, 2013, and 2014.

Year	Re	Ra	Raa	Rs	Rab	Rh
2012	2.16	1.80	1.83	2.65	2.17	3.45
2013	2.52	3.11	2.87	2.85	4.28	1.97
2014	2.69	3.43	4.74	2.11	2.55	1.77

Note: Values were calculated using data from July to September, as Ra and its components were extremely weak in May and June, and Ts had a minimal effect on them during these months (Figs 1 and 2).

We simulated the dynamics of Re and its components during the growing season with a multi-factor equation, Rx = a exp (b PAR + c Ts + d SWC + e GLAI) (Table 2). The goodness-of-fit values of the multi-factor equation were greater than 0.8 for Re, Ra, and Raa, indicating extremely good simulation results, but were relatively low for Rs, Rab, and Rh, mainly because these factors were disturbed by precipitation.

**Table 2:** Regression results of the multi-factor equation, Respiration = a exp (b PAR + c Ts + d SWC + e GLAI) for the growing seasons of 2012, 2013, and 2014. PAR is photosynthetically active radiation, Ts is the 5-cm soil temperature, SWC is the 10-cm soil water content, and GLAI is the green leaf area index. **Statistically significant at the p < 0.01 level (n = 153).

Rx	Year	a	b × 10^2^	c	d	e	R^2^
Re	2012	0.50	0. 08	0.08	0.28	0.27	0.89**
	2013	0.65	−0. 02	0.06	0.38	0.25	0.94**
	2014	0.90	0. 05	0.05	0.21	0.25	0.92**
Ra	2012	0.24	0. 18	0.06	1.47	0.40	0.89**
	2013	0.28	−0. 15	0.06	2.09	0.33	0.92**
	2014	0.30	0. 10	0.06	1.34	0.33	0.86**
Raa	2012	0.13	0. 03	0.06	2.38	0.42	0.81**
	2013	0.25	0. 05	0.03	1.37	0.44	0.92**
	2014	0.20	0. 42	0.04	1.55	0.42	0.81**
Rs	2012	0.47	0. 12	0.09	−2.59	0.18	0.62**
	2013	0.35	−0. 01	0.10	−0.79	0.08	0.67**
	2014	0.09	−0. 44	0.11	0.97	0.18	0.55**
Rab	2012	0.14	0. 42	0.06	−0.84	0.37	0.65**
	2013	0.20	0. 12	0.04	0.80	0.27	0.60**
	2014	0.04	−0. 37	0.11	4.09	0.20	0.55**
Rh	2012	0.35	0. 06	0.10	−3.28	0.04	0.50**
	2013	0.93	0. 62	0.05	−4.44	0.10	0.67**
	2014	1.52	0. 13	0.02	−2.54	0.09	0.51**

Note: Re: ecosystem respiration; Ra: autotrophic respiration; Raa: above-ground autotrophic respiration; Rs: soil respiration; Rab: below-ground autotrophic respiration; Rh: heterotrophic respiration.

#### Responses of Re and its components to GPP

The linear relationships between GPP and Re and its components were highly significant during the growing season in all three years (Table 3). The goodness-of-fit values between GPP and Re and its components were generally ranked in declining order from Re (from 0.83 to 0.88), to Ra, Raa, Rs, Rab, and finally to Rh (from 0.16 to 0.27).

**Table 3:** Linear relationships between gross primary production (GPP) and ecosystem respiration (Re), autotrophic respiration (Ra), above-ground autotrophic respiration (Raa), soil respiration (Rs), below-ground autotrophic respiration (Rab), and heterotrophic respiration (Rh) during the growing seasons of 2012, 2013, and 2014. Respiration = a GPP + b. **Statistically significant at the p < 0.01 level (n = 153).

Rx	Year	a	b	R^2^
Re	2012	0.33	1.65	0.83**
	2013	0.37	1.43	0.86**
	2014	0.29	1.96	0.88**
Ra	2012	0.34	—	0.72**
	2013	0.36	—	0.80**
	2014	0.30	—	0.81**
Raa	2012	0.22	—	0.57**
	2013	0.27	—	0.70**
	2014	0.20	—	0.72**
Rs	2012	0.15	1.19	0.53**
	2013	0.11	1.28	0.57**
	2014	0.10	1.74	0.57**
Rab	2012	0.12	—	0.60**
	2013	0.09	—	0.47**
	2014	0.10	—	0.33**
Rh	2012	0.04	0.93	0.16**
	2013	0.03	1.10	0.18**
	2014	0.03	1.26	0.27**

### Relationships between Re, Ra, and Rs and their components

There were highly significant linear relationships between Re, Ra, and Rs and their components during the growing season (Table 4). The goodness-of-fit value between Re and Ra was the highest, followed by that between Re and Raa. Although the linear relationship between Re and Rh was highly significant, the goodness-of-fit value was very low, which implies that Re and Rh followed different patterns. The changes in Ra and Raa followed similar patterns, as shown by the high goodness-of-fit value. The goodness-of-fit value between Rs and Rab was higher than that between Rs and Rh, which suggests that changes in Rab had a greater influence on Rs than Rh did.

**Table 4:** Linear relationships between totals and their components during the growing seasons of 2012, 2013, and 2014. Component = a Total + b. **Statistically significant at the p < 0.01 level (n = 153).

Total	Component	Year	a	b	R^2^
Re	Ra	2012	0.89	−0.90	0.92**
		2013	0.94	−1.12	0.97**
		2014	0.89	−1.08	0.97**
Re	Raa	2012	0.57	−0.58	0.76**
		2013	0.73	−1.03	0.90**
		2014	0.64	−1.03	0.86**
Re	Rab	2012	0.32	−0.33	0.77**
		2013	0.21	−0.09	0.54**
		2014	0.25	−0.05	0.59**
Re	Rs	2012	0.43	0.58	0.64**
		2013	0.27	1.03	0.56**
		2014	0.36	1.03	0.66**
Re	Rh	2012	0.11	0.90	0.13**
		2013	0.06	1.12	0.12**
		2014	0.11	1.08	0.30**
Ra	Raa	2012	0.65	—	0.91**
		2013	0.75	—	0.94**
		2014	0.67	—	0.90**
Ra	Rab	2012	0.35	—	0.70**
		2013	0.25	—	0.53**
		2014	0.33	—	0.53**
Rs	Rab	2012	0.59	−0.36	0.74**
		2013	0.69	−0.68	0.77**
		2014	0.66	−0.69	0.82**
Rs	Rh	2012	0.41	0.36	0.58**
		2013	0.31	0.68	0.40**
		2014	0.34	0.69	0.55**

Note: Re: ecosystem respiration; Ra: autotrophic respiration; Raa: above-ground autotrophic respiration; Rs: soil respiration; Rab: below-ground autotrophic respiration; Rh: heterotrophic respiration.

During the growing season, Re ranged from 660 to 740 g C m^−2^; Ra accounted for between 64% and 71% of Re, and Raa accounted for between 63% and 73% of Ra. Values of Rs were between 357 and 394 g C m^−2^ during the growing season, of which Raa accounted for between 39% and 44% (Table 5).

**Table 5:** Comparisons of ecosystem respiration (Re), the ratio of autotrophic respiration (Ra) to Re (Ra/Re), the ratio of soil respiration (Rs) to Re (Rs/Re), Ra, the ratio of above-ground autotrophic respiration (Raa) to Ra (Raa/Ra), Rs, the ratio of below-ground autotrophic respiration (Rab) to Rs (Rab/Rs), and heterotrophic respiration (Rh) for different vegetation types under different climates.

Site	Latitude	Method	Period	Vegetation	Re	Ra/Re	Rs/Re	Ra	Raa/Ra	Rs	Rab/Rs
					gC m^−2^	%	%	gC m^−2^	%	gC m^−2^	%
Central Italy^32^	N43°44′	EC + SR + TG	16 May–31 Oct 2011	Mediterranean pine	1045	62	53	645	77	550	27
Vancouver Island, Canada^31^	N49°52′	EC + SR + RE	1 Jan–31 Dec 2005	Douglas fir	1621	63	61	1026	61	994	40
Xinjian, China^5^	N28°54′	SG + RE	2 Oct 2010–16 Jun 2011	Carex	862	62	64	535	58	552	41
Xilin Haote, China^43^	N43°38′	SG + RE	26 Jul–31 Aug 2008	Leymus chinensis	—	53	59	—	77	—	20
Haibei, China^42^	N37°36′	SG + RE	1 Jul–30 Sep 2003	Kobresia humills	384	55	—	—	—	—	—
			1 Jul–30 Sep 2003	Potentilla fruticosa	466	63	—	—	—	—	—
Lonzée, Belgium^2^	N50°33′	EC + SR + RE	2 May–8 Aug 2006	Potato	273	67	47	184	78	128	30
			2 Apr–19 Jul 2007	Winter wheat	692	79	38	547	78	263	45
			15 May–18 Sep 2008	Sugar beet	582	62	63	362	59	368	40
Luancheng, China^21^	N37°53′	EC + SR + BI	17 Oct 2007–10 Jun 2008	Winter wheat	692	65	41	447	92	281	13
			11 Jun–2 Oct 2008	Summer maize	841	53	49	444	96	413	4
Qiyang, China^18^	N26°45′	SG + RE	31 Mar–17 Jul 2009	Spring maize^a^	—	—	—	—	—	428	35
			31 Mar–17 Jul 2009	Spring maize^b^	—	—	—	—	—	185	44
			31 Mar–17 Jul 2009	Spring maize^c^	—	—	—	—	—	100	40
Wageningen, Netherlands^20^	N51°59′	EC + SR + BI	15 May–9 Oct 2007	Spring maize	1197	84	41	1004	70	492	61
Shouyang, China	N37°45′	EC + SR + RE	1 May–30 Sep 2012	Spring maize	671	69	56	462	64	370	44
			1 May–30 Sep 2013	Spring maize	740	71	48	524	73	357	39
			1 May–30 Sep 2014	Spring maize	660	64	60	421	63	394	39

Note: EC: eddy-covariance system; SR: automated soil respiration system; TG: tree girdling method; RE: root exclusion method; SG: static chamber-gas chromatography techniques; BI: biomass investigation method.

^a^210 tN ha^−1^, 37 tP ha^−1^, 73 tK ha^−1^ and straw return; ^b^210 tN ha^−1^, 37 tP ha^−1^, 73 tK ha^−1^; ^c^without fertilizer^18^.

**Figure 4:**
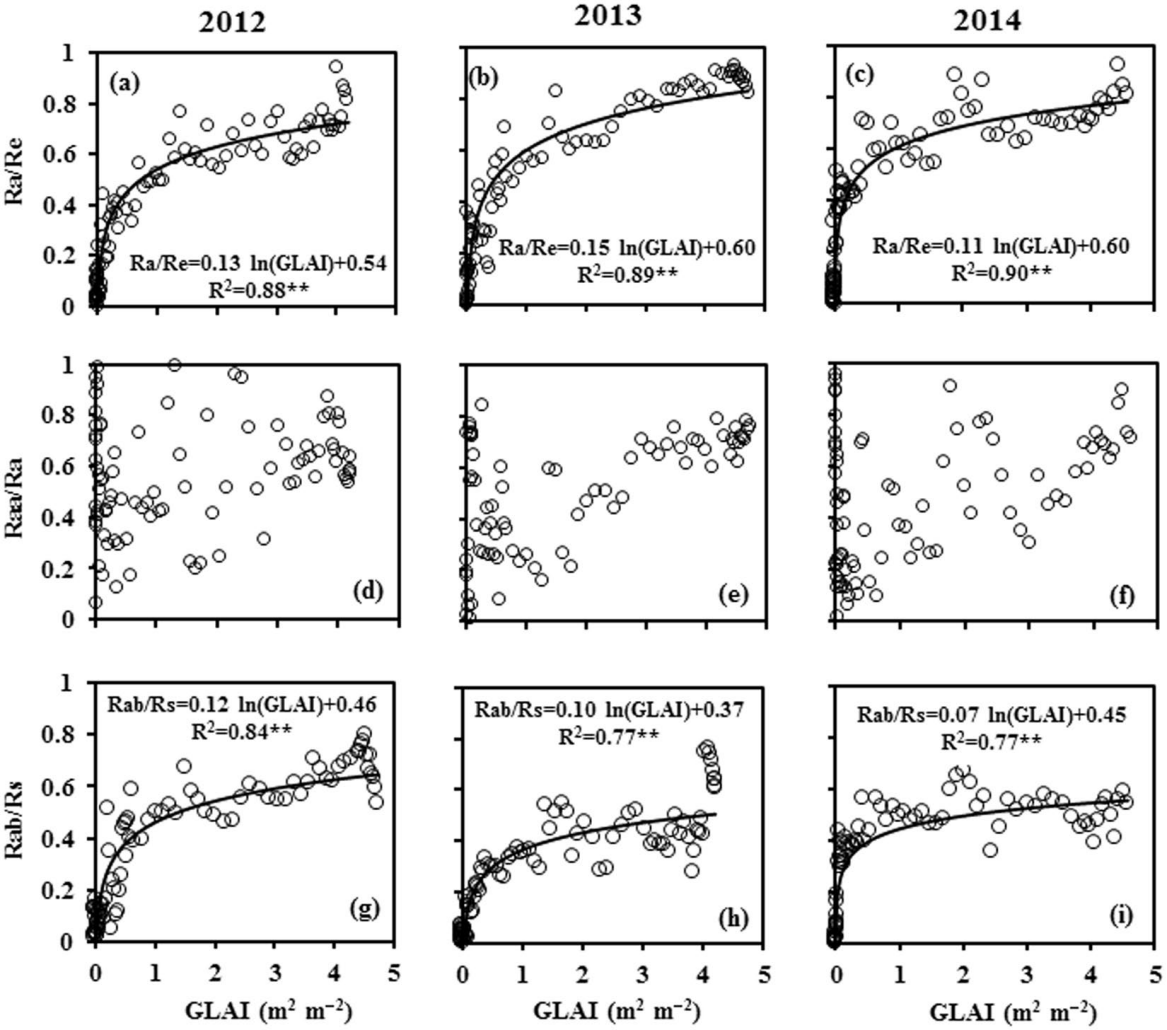
Relationship between the green leaf area index (GLAI) and each of the ratio of autotrophic respiration to ecosystem respiration (Ra/Re), the ratio of above-ground autotrophic respiration to autotrophic respiration (Raa/Ra), and the ratio of below-ground autotrophic respiration to soil respiration (Rab/Rs) during the growing seasons of 2012, 2013, and 2014. All data in the figure were obtained during the period before GLAI reached its maximum, because of uncertainties in the measuring method. **Statistically significant at the p < 0.01 level (n = 97, 97, and 93 in 2012, 2013, and 2014, respectively).

We used a simple logarithmic equation (Ra/Re and Rab/Rs = a ln (GLAI) + b) to describe the relationships between Ra/Re and GLAI and between Rab/Rs and GLAI (Fig. 4). When GLAI was below 1 m^2^ m^−2^, Ra/Re and Rab/Rs increased sharply and then increased slowly as GLAI increased further. There was no clear relationship between Raa/Ra and GLAI. When GLAI was relatively small, the data points of Raa/Ra were significantly dispersed because Raa and Ra were also small, but the data points became increasingly concentrated as GLAI increased.

## Discussion

### Effect of environmental factors on Re and its components

The main control on photosynthesis, PAR^20,33^, is an important influence on Ra, Rs, and Re because it provides the resources for respiration^32^. In view of this, tree girdling has been widely used to partition Rs into Rh and Rab in forests^32^. Studies reported decreases in Rs of 53% after four days of shading^34^, and showed that solar radiation is an important control on Rs in maize farmland^35^ and grassland^36^. In the present study, PAR had a positive effect on Re (2013 and 2014), Ra (2014), Rab (2012), and Rh (2013) (Fig. 3), which implies those respirations may decrease in spring maize croplands in the Loess Plateau as dimming develops further^30^. However, PAR had a relatively weak effect on Re and its components in our study, and we suspect that the Lloyd & Taylor equation, used to estimate the daytime Re, may have concealed the effect of PAR on Re and its components to some degree.

Air temperature and Ts are the indexes most commonly used to determine the relationships between temperature and Re and its components, and Ts is generally consistent with air temperature in agro-ecosystems^24^. Respiration is essentially a series of enzymatic reactions, meaning that temperature is positively correlated with Re and its components. In this study, the partial correlation coefficients between Re and its components and Ts ranged from 0.25 to 0.81. However, when very high or low SWCs are the main constraint on Rs and its components, the positive correlation between temperature and Rs and its components may weaken or even change direction^8,25^. As in this study, other studies have reported differences among the Q_10_ values of Re and its components^2,5,7,24^ (Table 1), which implies that Re and its components behave differently under warming at our site. There has been much discussion about the Q_10_ of Re, especially the differences among its components. The Q_10_ of Rh may be greater^2^ or less^5,7,24^ than that of Ra and its components in various ecosystems. In our study, the Q_10_ values of Rh were higher than those of Ra in 2012, and lower in 2013 and 2014 (Table 1), probably reflecting the obvious priming effect of precipitation on Rh in July 2012, which resulted in a high Q_10_ for Rh. This result indicates that we should measure Re and its components over several years, because the Q_10_ value of Ra may be higher or lower than that of Rh in different years in the same ecosystem. In general, the three-year average Q_10_ value of Ra was higher than that of Rh, indicating that Ra was more sensitive to global warming than was Rh at our study site.

In rainfed agricultural regions, SWC is an important control on crop growth^28^, and it generally promotes Ra, Raa, and Re in this and other previous study^37^. When the soil is dry, Rs and its components increase sharply after rainfall as SWC increases, referred to as the priming effect of precipitation^20,25^. The carbon emissions that result from this priming effect have an important influence on the total Rs in dry areas^38^. Still, when the soil is wet, the gas exchange between the air and the soil is inhibited by the excessive soil water after rainfall. The Rs and its components generally decrease with SWC, and when SWC exceeded 0.27 m^3^ m^−3^ at the study site, Rh was more sensitive to SWC than was Rab^8^. The critical point of SWC when Rh decreased with SWC was lower at our site than in other studies^24^, perhaps because the soil organic matter was relatively low and the soil porosity was poor. The relationships between SWC and Rs and its components were different for each of the three years because of the combined effects of increases in SWC on Rs and its components. The average daily rainfall intensity has decreased significantly in the Loess Plateau^29^, and this may weaken the inhibitory effect of excessive soil water on Rs and its components in the future.

Green leaf area index is an important control on Ra and its components, and on Re and Rs. Because the green leaf area is the effective area for photosynthesis and Raa, it also influences the development of living roots, where Rab is effective. Therefore, the SWC, temperature, and PAR will affect Ra and its components by acting on green leaves or living roots. The partial correlation coefficients between GLAI and Re and its components ranged from 0.23 to 0.95 in our study. The number of consecutive dry days has significantly increased in the Loess Plateau^29^, which may result in poor plant establishment that in turn will cause Re and its components to decrease in the future. Agricultural management practices such as plastic film mulching and fertilizer usually promote crop growth and result in increases in Re and its components. However, GLAI is not generally used as an environmental variable to fit the Re curve in natural ecosystems^37,39^, mainly because the seasonal variations in GLAI and air temperature in temperate regions are similar, and GLAI is relatively stable in evergreen forests during the growing season. Agricultural ecosystems are characterized by rapid vegetation development during the growing season and are strongly influenced by human activities. Usually, GLAI is an important control on Re in agricultural ecosystems. For example, an exponential relationship was reported between nighttime Re and GLAI in maize and soybean farmlands^33^. In tropical areas, a linear relationship was reported between Re and GLAI in maize and rice farmlands^19^. Consistent with these earlier results, there was a highly significant relationship between Re and GLAI in our study, with partial correlation coefficients between Re and GLAI of 0.91, 0.94, and 0.94 during the growing seasons of 2012, 2013, and 2014, respectively.

### Effect of GPP on Re and its components

Gross primary production is used as a surrogate for processes related to the total carbon uptake of crops and serves as a direct indicator of photosynthesis; hence, Ra and its components are controlled by GPP even though there is a delay between assimilation and photoassimilate consumption in the development process^2^, which is 2 hours for maize^24^. For annual crop plants, the regression lines between GPP and Ra and its components (Table 3) and between Ra and its components (Table 4) should pass through zero, because they are derived from living plants. Slopes of 0.3 between GPP and Ra were reported for winter wheat, sugar beet, and potatoes in Belgium^40^, which are comparable to the slopes for spring maize in the present study; however, another study reported much lower slopes between GPP and Ra and its components for the same three crops in Belgium^2^, perhaps because Ra and its components were standardized at 10 °C. In the latter part of the crop growing season, the slope between GPP and Ra increased^40^, reflecting the fact that Ra included respiration of dead leaves and roots, which cannot be avoided when the eddy covariance data and soil chamber measurements are combined. The slope of the regression line between GPP and Raa was significantly larger than that between GPP and Rab in this and other previous studies, because the values of Raa were much higher than those of Rab^2^. A previous study reported a slope value of only 0.04 between GPP and Rab for summer maize in the North China Plain^24^, which is much lower than the corresponding value in our study and mainly reflects the relatively larger intercept of the linear equation in the earlier study.

Gross primary production and Re are usually controlled by the same factors, and Ra dominates Re in croplands characterized by rapid vegetation development^2,20^. Consistent with our results, other studies have reported a linear relationship between GPP and Re in agricultural ecosystems^19,37^. In the present study, the dynamics of Rs were controlled by Rab, and GPP had a distinct influence on Rs. The highly significant linear relationship between GPP and Rs in this study (Table 3) suggests that it may be possible to estimate GPP from Rs in field plot experiments where it is relatively difficult to measure GPP directly. Because Rh results mainly from residue and soil organic carbon decomposition, which should be independent of GPP on a daily to monthly time scale, Rh was less sensitive to GPP than Re and its other components were^2^. This is not necessarily true over long time scales, because the material for Rh is dependent on the GPP of the previous years in non-managed ecosystems^41^. However, in agricultural ecosystems there may not be a clear relationship between GPP and Rh over long time scales, because the material for Rh may be from carbon inputs, such as manure and crop residues. Although carbon inputs usually cause increases in Re and its components by promoting crop growth and providing the material for Rh, they are ultimately beneficial for carbon sequestration in agricultural ecosystems.

### Characteristics of Re and its components

In the present study, Re reached maximum values of 9.45, 10.63, and 9.58 g C m^−2^ d^−1^ in 2012, 2013, and 2014, respectively. These values are relatively low compared to those reported in previous studies; for example, Re maximum values of between 13.00 and 16.60 g C m^−2^ d^−1^ were reported for spring maize fields^20,33^. At our study site, Re averaged 690 g C m^−2^ during the growing season, which is lower than the values reported previously for spring and summer maize^17,20,21^, and this result reflects the climate of the plateau, the large diurnal ranges and seasonal variations in temperature, and the low content of soil organic matter. The maximum Re of spring maize at the study site was generally higher than that reported for winter wheat, sugar beet, and potato^2,21^, higher than that for grasslands^42,43^, and similar to that reported for some forests^31,32^, which indicates that physiological activity in spring maize during the vigorous growth stage at our site was stronger than in other crops.

The ratios of Ra to Re in the present study were 69%, 71%, and 64% in 2012, 2013, and 2014, respectively, which suggests that Re is dominated by Ra in the growing season, and that vegetation development has a greater influence on Re than do other abiotic variables in this spring maize field. The values of this ratio in our study are higher than those reported for grasslands on the Inner Mongolian Plateau^43^ and the Qinghai–Tibetan Plateau^42^, where the grassland ecosystems are severely degraded. In addition, the values are lower than those reported for a winter wheat field during the vigorous growth stage in Belgium^2^ and for a spring maize field with strong physiological activity in the Netherlands^20^, and comparable to those reported in other studies in a range of ecosystems, as listed in Table 5. The R^2^ values between the Ra/Re and GLAI in our study were 0.88, 0.89, and 0.90 in 2012, 2013, and 2014, respectively (Fig. 4), indicating that GLAI can be used to partition Re into Ra and Rh. Given that GLAI has been used to partition evapotranspiration into soil evaporation and transpiration^22^, we believe that it can also be used to partition Re into Ra and Rh in croplands.

The Rs-to-Re ratios of 56%, 48%, and 60% calculated for 2012, 2013, and 2014, respectively, are comparable to those reported in most of the studies cited in Table 5. Our ratio values are only higher than those for some winter wheat^2,21^ and spring maize^20^ fields, perhaps because the physiological activity of the above-ground plants was somewhat stronger than that of the soil roots and microorganisms in the earlier studies. The linear relationship between Re and Rs in this study is highly significant (Table 4), and R^2^ values were 0.64, 0.56, and 0.66 in 2012, 2013, and 2014, respectively, so it might be possible to estimate Re from Rs in field plot experiments where is it difficult to measure Re directly.

We observed growing season Ra values of 462, 524 and 421 g C m^−2^ in 2012, 2013, and 2014, respectively. These values are lower than the value of 1004 g C m^−2^ observed for spring maize in the Netherlands^20^, where the mean annual temperature and precipitation were higher than in the Loess Plateau and crop growth was promoted by high levels of soil organic matter. The Ra values for the present study site are lower than those for ecosystems with longer growing seasons, such as evergreen forests^31,32^ and a meadow on a lake shore^5^ (Table 5). The values are comparable to those for winter wheat and summer maize^2,21^ grown in other areas with different local climate conditions and soils, and higher than those for sugar beet and potatoes grown in areas with shorter growing seasons^2^.

The ratios of Raa to Ra in our study were 64%, 73%, and 63% in 2012, 2013, and 2014, respectively, indicating that Ra was dominated by Raa in the growing season and that canopy development controlled Ra in our spring maize field. The ratios in the present study are higher than those reported for a sugar beet field^2^ and a meadow on a lake shore^5^, possibly because the root systems of these two vegetation types were more developed. The ratios are lower than those reported for a cropland in the North China Plain where winter wheat and summer maize were rotated^21^, possibly because Rab was underestimated by the method used in the earlier study. Otherwise, the present values are comparable to those reported in other studies, as listed in Table 5. Although the relationship between Raa/Ra and GLAI was subtle, Raa/Ra generally increased with GLAI (Fig. 4), which implies that an increasing amount of photoassimilates was distributed to the above-ground plants as they grew in our spring maize field.

We observed Rs values of 370, 357, and 394 g C m^−2^ in the growing seasons of 2012, 2013, and 2014, respectively, lower than the values previously observed over longer periods and in soils with higher organic matter in evergreen forests^31,32^ and in a meadow on a lake shore^5^ (Table 5). The Rs values measured in our study site are higher than those measured over shorter periods in winter wheat and potato fields in Belgium^2^ and in a winter wheat field under lower temperatures in the North China Plain^21^, but are comparable to those measured in a sugar beet field in Belgium^2^. Compared with the Rs values for our study site, those for maize fields under similar agricultural management show higher values^18,20^, reflecting higher temperatures or more developed root systems in the earlier studies. And the growing-season Rs values for spring maize fields without straw return or fertilizer^18^ are lower than those for our study site. Straw return and fertilizer supply large amounts of material for Rh and generally promote crop growth^18,26^, resulting in increases in Rab and Rs.

We calculated Rab-to-Rs ratios of 44%, 39%, and 39% for 2012, 2013, and 2014, respectively, indicating that the quantity of Rs in our study was controlled by microbial activity, whereas changes in Rs were controlled mainly by Rab (Table 4). The Rab-to-Rs ratios were higher than those calculated for a Mediterranean pine forest^32^, a *Leymus chinensis* grassland^43^, and a potato field^2^, where microbial activity was stronger than root activity. The Rab-to-Rs ratios for a winter wheat field and a summer maize field^21^ were only 13% and 4%, respectively, because Rh was very low, only 36 and 16 g C m^−2^ for the winter wheat field and summer maize field, respectively. The Rab-to-Rs ratio was 67% in a spring maize field^20^ where root activity were stronger than microorganism activity. The ratios obtained in the present study are comparable to those reported in other studies cited in Table 5. The R^2^ values between Rab/Rs and GLAI in our study were 0.84, 0.77, and 0.77 in 2012, 2013, and 2014, respectively (Fig. 4), indicating that GLAI can be used to partition Rs into Rab and Rh.

## Conclusions

Our three-year study of the growing season revealed seasonal patterns in Re and its components in a rainfed spring maize field, and also determined the relationships between, and controls on, Re and its components. Ecosystem respiration and its components (apart from Rh) generally increased early in the growing season and then decreased. A multi-factor equation (Rx = a exp (b PAR + c Ts + d SWC + e GLAI)) could be used to describe the seasonal variations in Re and its components, especially those in Re, Ra, and Raa. The highly significant linear relationships between GPP and Re and its components suggest that the latter are driven by GPP. The linear relationships between Re, Ra, and Rs and their components are also highly significant. We found that Re was dominated by Ra, and Ra was dominated by Raa. Although Rh was the main component of Rs, the changes in Rs and Rab were more similar to each other. The relationships between GLAI and each of Ra/Re and Rab/Rs can be described by a simple logarithmic equation, which indicates that Ra/Re and Rab/Rs are controlled by GLAI. In croplands, Re and its components are sensitive to management practices, and future studies should examine how Re and its components respond to different management practices and climate change.

## Materials and Methods

### Site description

We carried out our experiments over three consecutive growing seasons from 2012 to 2014 in the eastern part of the Loess Plateau in Shanxi Province, China, at the Ministry of Agriculture’s Shouyang Dryland Farming Experimental Station (37°45′N, 113°12′E, 1202 m a.s.l.). The experimental site has a semi-arid temperate continental monsoon climate with a prolonged cold and dry winter and a short and hot rainy summer. The mean annual precipitation is 474.5 mm, with more than 70% of this total falling between July and September. The mean annual temperature is 8.2 °C and the mean annual frost-free period is 150 days. The soil organic matter, total nitrogen, total phosphorus, and total potassium concentrations of top soils at the site were 9.00, 0.79, 0.72, and 19.61 g kg^−1^, respectively.

Crops in this region are dominated by single-crop rainfed spring maize, the growing season for which starts on May 1 and ends on September 30. For our study site, we chose a rainfed spring maize cropland (100 × 260 m) that was cultivated and managed in line with the practices of local farmers. The spring maize on our site was sown around May 1, with 50 cm between rows and 30 cm between plants. Before the maize was sown, nitrogen (N), phosphorus (P), and potassium (K) fertilizers were applied to the field as a basal dressing at the rates used by local farmers (207 kg N ha^−1^, 47 kg P ha^−1^, and 37 kg K ha^−1^) and were completely mixed with the soil during tillage in late April. The spring maize matured before 30 September. Straw was chopped with an automated machine and returned to the field at harvest time.

### Eddy flux measurements

Carbon fluxes were measured continuously with an open-path eddy covariance system set up in the center of the maize field. The fetch of the prevailing wind was about 140 m, which satisfied the measurement requirements. The eddy-covariance system consisted of a three-dimensional sonic anemometer (CSAT3, Campbell Scientific Inc., Logan, UT, USA) and an open-path infrared gas analyzer (LI-7500, Li-COR Inc., Lincoln, NE, USA) positioned at a relative height of almost 1.3 m above the crop canopy. All data were collected at 10 Hz with a data logger (CR5000, Campbell Scientific Inc.) and were block-averaged at 30-min intervals for analyses and archiving.

### Soil respiration measurements

We used an automated soil CO_2_ flux system (LI-8100, Li-COR Inc.) connected to six long-term dark chambers (LI-8100-104, Li-COR Inc.) with a multiplexer (LI-8150, Li-COR Inc.) to measure Rs and Rh during the spring maize growing seasons of 2012, 2013, and 2014. The plastic collars of the chambers (20.3 cm in inner diameter, 11 cm in height) were put in place after tillage. Heterotrophic respiration was measured in three randomly selected chambers using three home-made stainless-steel frames (0.4 × 0.4 × 0.4 m), as outlined in the root exclusion method^5^. These three frames were reinstalled each year after tillage. Soil respiration was measured in the remaining chambers. Measurements were collected automatically from each chamber for 4 minutes at 1-hourly intervals.

### Auxiliary measurements

A rain gauge (TE525, Texas Electronics Inc., Dallas, TX, USA) and a PAR sensor (LI190SB, Li-COR Inc.) were connected to a data logger (CR3000, Campbell Scientific Inc.) that collected data every 30 minutes. We connected six soil moisture sensors (8150-202, Li-COR Inc.) and six soil temperature sensors (8150-203, Li-COR Inc.) to the chambers to measure SWC and Ts. During the growing season, seven spring maize plants were randomly selected to manually measure the green leaf length and maximum width every 6 to 10 days after the seedlings emerged. We calculated GLAI using a method proposed previously^44^.

### Data processing

EddyPro software (Version 5, Li-COR Inc.) was used to calibrate and quality control the 10 Hz carbon fluxes data from the eddy covariance system. The software produced data at 30-min intervals. We rejected the data if less than 70% of the 30-min flux footprint overlapped with the area of interest^21^ or if the friction velocity was less than 0.15 m s^−1^ at night^45^. We used linear interpolation to fill short data gaps (≤2 h), and the Michaelis–Menten^46^ and Lloyd & Taylor^47^ equations to fill long data gaps (>2 h) during the daytime and nighttime, respectively^47^. We also used the Lloyd & Taylor equation to estimate the daytime Re. Once these steps were completed, we then calculated GPP^20^.

We calculated Ra, Raa, and Rab from Re, Rs, and Rh, as follows:

1 $$Ra={Re}-Rh$$

2 $$Raa={Re}-Rs$$

3 $$Rab=Rs-Rh$$

The temperature sensitivity of Re and its components was calculated as follows:

4 $${R}_{x}=\alpha \,\exp (\beta {T}_{s})$$

5 $${Q}_{10}=\exp (10\beta )$$

where *Rx* represents Re and its components, *α* and *β* are parameters, *Ts* is the 5-cm soil temperature, and *Q*_10_ is the temperature sensitivity.

Rh was calibrated as follows^2^:

6 $${R}_{h}^{^{\prime} }={R}_{h}{Q}_{10}^{(T^{\prime} -T)/10}$$

where *R*′*h* is the temperature-calibrated Rh, *Q*_10_ is the soil temperature sensitivity of Rh, and *T′* and *T* are 5-cm soil temperatures inside and outside of the stainless-steel frames, respectively.

### Statistical analyses

All statistical analyses were performed with SPSS for Windows (Version 18, SPSS Inc., Chicago, IL, USA). We used simple linear regression to evaluate the relationships between Rs and Re and between Rs and Rh before the seedlings emerged, between GPP and Re and its components, and between Re, Ra, and Rs and their components. We tested the influence of PAR, Ts, SWC, and GLAI on Re and its components with partial correlation analysis. We evaluated the relationships between Re and its components and PAR, Ts, SWC, and GLAI with multiple nonlinear regression. The relationships between GLAI and Ra/Re, Raa/Ra, and Rab/Rs were evaluated with logarithmic regression.

### Uncertainties in the methods

**Figure 5:**
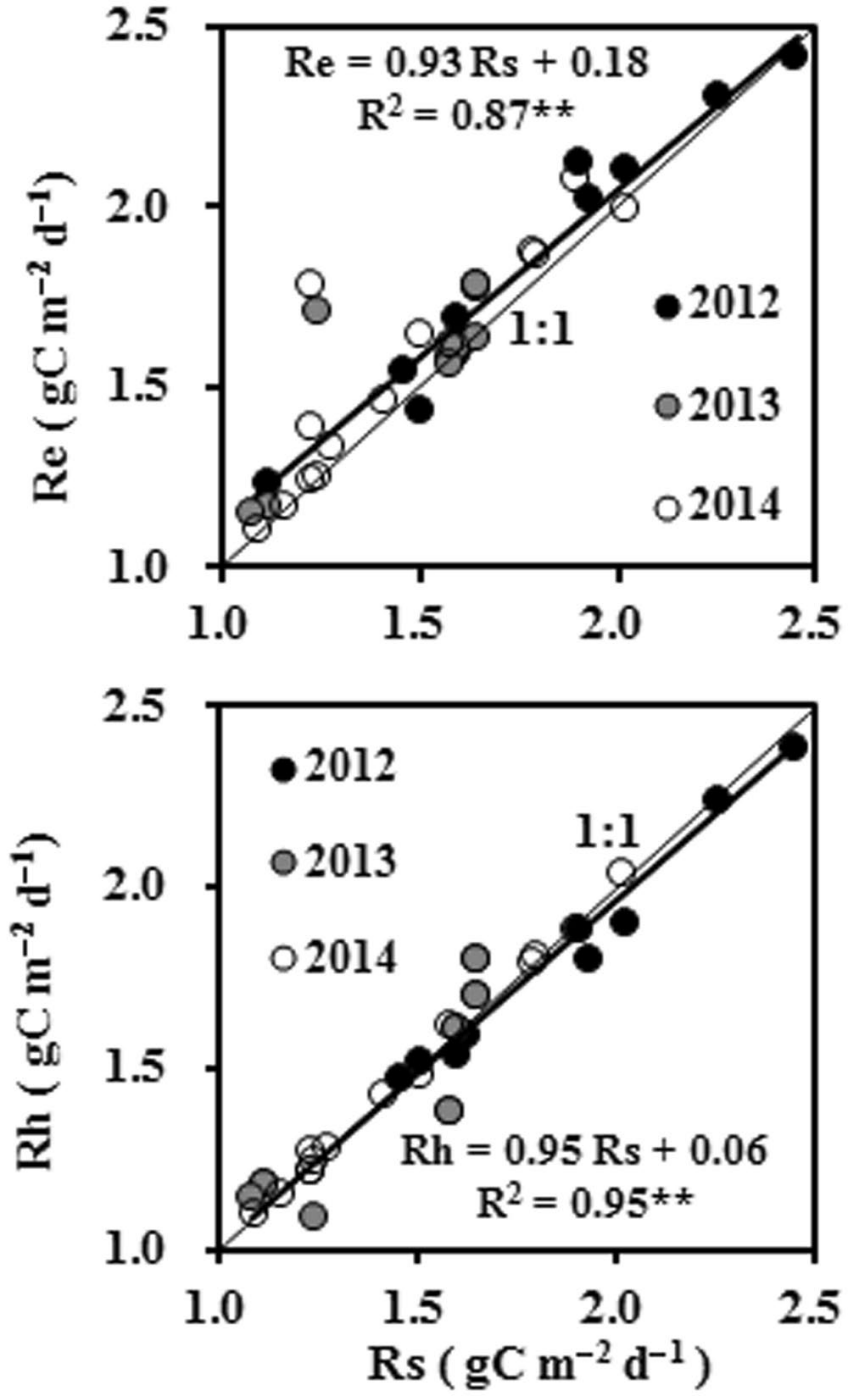
Comparison between soil respiration (Rs) and (**a**) ecosystem respiration (Re) and (**b**) heterotrophic respiration (Rh) before seedlings emerged during the growing seasons of 2012, 2013, and 2014. **Statistically significant at the p < 0.01 level (n = 35).

In this study, the relationship between Rs and Re was expressed by a well-fitted linear curve with a goodness-of-fit value of 0.87 for the period before the spring maize seedlings emerged, and all data points were close to the line 1:1 in 2012, 2013, and 2014 (Fig. 5a). This shows that our method (i.e. using the eddy covariance technique combined with soil chamber measurements) was suitable for studying Re and its components at our study site. This method, however, has some disadvantages. For example, although Rs was consistent with Rh during the period before seedlings emerged (Fig. 5b), the root exclusion method introduced uncertainty to the discrimination between Rh and Rab. This was mainly because (1) the soil in the home-made frame did not receive organic carbon inputs from root exudation, which contributed to the priming effect^2^; and (2) the soil moisture patterns within the home-made frame were different from those in the uncontrolled area^24^, because soil water movement was restricted by the frame. The method we used to fill the gaps in the eddy covariance measurements was another source of uncertainty^2^. In addition, Raa included dead leaf respiration, especially after GLAI reached its maximum value, and the similar situation also existed in the measurement of Rab. Therefore, calculations of Ra were also affected by various uncertainties after GLAI reached its maximum value.
